# Does norovirus induce acute hepatitis?

**DOI:** 10.3934/publichealth.2020013

**Published:** 2020-03-09

**Authors:** Carmen Lok Tung Ho, Olivia Oligbu, Fatma Asaid, Godwin Oligbu

**Affiliations:** 1 Imperial College School of Medicine, London, UK; 2 Department of Paediatrics, Dr Gray's Hospital, NHS Grampian, Elgin, Scotland, UK; 3 Paediatric Infectious Diseases Research Group, Institute for Infection and Immunity, St. George's, University of London, UK

**Keywords:** norovirus, acute hepatitis, transaminitis, gastroenteritis, liver

## Abstract

**Background:**

Norovirus is the commonest cause of acute viral gastroenteritis with significant morbidity. Extra intestinal manifestation following norovirus infection is rare and the mechanism is unknown.

**Methods:**

We undertook a review of the English literature published from January 1967 to April 2019 to evaluate the risk of acute viral hepatitis due to norovirus gastroenteritis. Data sources included MEDLINE, EMBASE, Cochrane library, and references within identified articles.

**Results:**

We identified 126 potential studies and included 5 publications involving 17 cases of norovirus induced hepatitis, and all had elevated ALT (31.7–458IU/l) and AST levels (45.6–1150IU/l). Majority of the cases were below the age of 18 (88%, n = 15) and almost two-third (64.7%, n = 11) had supportive treatment, mainly intravenous fluid administration. In cases reporting sex, there were more females than males (62.5%, 5/8 vs. 37.5%, 3/8). The duration of illness was longer, on average 10 days, compared to 3 days in those without elevated transaminitis and it took an average of 22.5 days for liver enzymes to settle. All patients recovered fully with no progression to chronic liver disease.

**Conclusion:**

Norovirus gastroenteritis is a self-limiting illness with majority not requiring hospitalisation and invasive investigations. We recommend that clinicians should be aware of norovirus induced transaminitis, and to suspect this especially in children who are likely to have protracted illness and require hospitalisation due to norovirus acute hepatitis.

## 1. Introduction

Human norovirus is an RNA virus of the family Caliciviridae, and a leading cause of acute viral gastroenteritis, resulting in significant morbidity worldwide [1],[2]. In the United States, it has been estimated that there are 5.5 million cases of norovirus gastroenteritis per year, which represents 58% of cases of acute gastroenteritis [3]. Infection with norovirus typically causes nausea, vomiting, diarrhoea and abdominal pain. The symptoms usually develop 12 to 48 hours after infection, and on the average could last between 1 to 3 days. However, for some individuals, especially children, the elderly and individuals with underlying diseases, these symptoms can be severe and lead to dehydration, and in very rare cases even cause death [3],[4].

For decades, norovirus infection has not been associated with extra intestinal manifestation, particularly causing liver injury. Although, hepatotropic viruses, such as hepatitis A through to E, are the most common viral cause of acute hepatitis, other viruses such as rotavirus, Epstein-Barr virus (EBV), herpes simplex virus (HSV), cytomegalovirus (CMV), and varicella zoster virus (VZV) can also cause acute liver injury [5]. However, there has been recent reports of extra intestinal disease following norovirus infection [6]–[8], but little is known of norovirus association with acute hepatitis. We, therefore, review all published studies to evaluate the clinical presentation, management and outcomes of acute hepatitis following norovirus acute gastroenteritis. It is hoped that the findings of this review will provide clinicians with a robust evidence base to investigate and manage patients suspected with norovirus induced hepatitis.

## 2. Methods

### 2.1. Information sources and search strategy

A search strategy was designed to identify studies reporting elevated transaminitis (acute hepatitis) following norovirus acute gastroenteritis. We searched MEDLINE, EMBASE, and the Cochrane library from 01 January 1967 to 15 April 2019. The medical subject headings (MeSH) terms used included “hepatitis”, “transaminitis”, “liver function test”, “transaminases”, “liver”, “norovirus”, “norovirus-induced” and “Norwalk”. These MeSH terms were used in different combinations. The primary search strategy was “norovirus” AND (“hepatitis” OR “transaminitis” OR “transaminase” OR “liver”). We only included studies published in the English language in our review. In addition, we screened reference lists of selected papers to retrieve relevant studies. Appendix 1 shows the full search strategies used.

### 2.2. Selection of studies

Inclusion criteria required the study to report norovirus induced hepatitis or transaminitis, which was defined as evidence by elevated transaminitis (Aspartate transaminase +/− Alanine aminotransferase). Studies were excluded if they were studies not written in the English language, laboratory, experimental, animal or not original studies. Two independent reviewers (C.H. and G.O.) screened the title and abstract of papers identified by the electronic searches, evaluating inclusion and exclusion criteria for all papers. We retrieved full articles of included publications and each publication was then independently reviewed for eligibility.

### 2.3. Quality assessment and data extraction

Two reviewers (C.H. and G.O.) independently reviewed the methodological quality of included studies, comparability of case and controls, and outcomes. Discrepancies were resolved by discussion with a third author (F.A.). The specific variables extracted from the publications included: study design, country, age of participants, year of study, method of data collection, method of diagnosis of norovirus, definition of hepatitis, whether other causes of hepatitis, such as EBV and CMV, were excluded, clinical presentation, liver function test (LFT) results, other abnormal test results, duration of illness, management, any previous medical issues and outcomes. The study quality assessment for reporting systematic reviews was done according to the Preferred Reporting Items for Systematic Reviews and Meta-analyses (PRISMA) statement [9].

### 2.4. Data analysis

All studies included in the review were summarised using descriptive analyses to provide an overview of the information on norovirus induced hepatitis, clinical presentations, management, complications and outcome.

**Figure 1.**
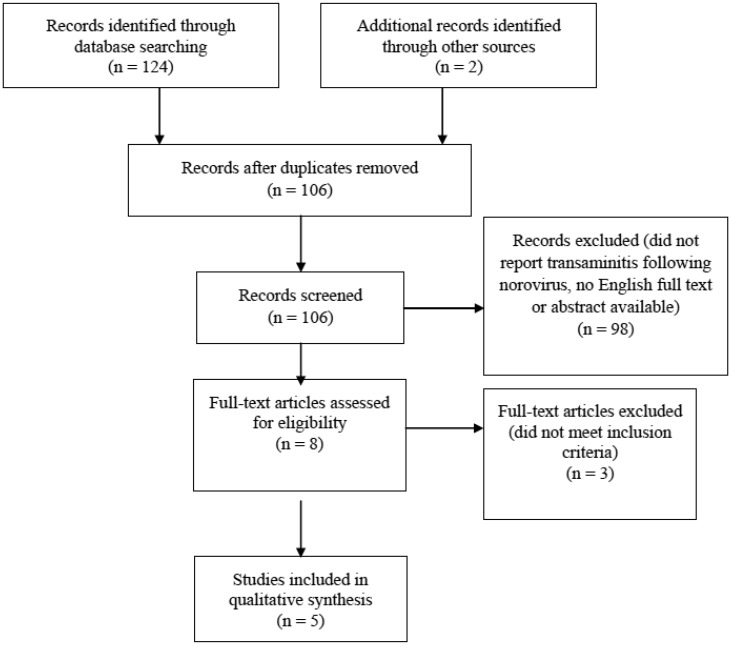
PRISMA flow diagram demonstrating identification and selection of eligible studies in the systematic review.

## 3. Results

### 3.1. Study characteristics

We identified 126 potential studies during the initial search, of which 20 were duplicates. Of the remaining 106 studies, 98 studies were excluded on the basis of title and abstracts, and a further 3 articles did not meet the inclusion criteria (Figure 1). Kucuk et al. [10] investigated all cases of transaminitis following viral gastroenteritis but we were able to extract data on norovirus associated transaminitis. Of the included studies, all cases were identified using routine investigations. Five studies [6],[10]–[13] were eligible for inclusion in the final analysis. Most articles were published from Japan (60%, n = 3/5), and one study each from Turkey and USA respectively. Out of the five studies, 2 were case reports, 2 were case series and the remaining one was a retrospective hospital-based study. Three articles involved patients who were younger than 18 years old [10],[12],[13] and the remaining two articles described patients older than 18 years [6],[11]. Three articles had a detailed description of the patient's past medical history [6],[11],[13]. Two of the five articles [11],[13] mentioned using stool specimens for norovirus antigens and three articles used polymerase chain reaction (PCR) [6],[10],[12]. A third diagnostic method, used by two of the articles, were rapid antigen tests [6],[10]. All articles stated that they excluded other possible causes of hepatitis and all included the levels of aspartate transaminase (AST) and alanine aminotransferase (ALT) measured. A summary of the study design, data collection method, study subjects, definition of hepatitis, management and outcome is presented in Tables 1 and 2.

There was a total of 17 cases of norovirus induced hepatitis, and all had elevated ALT (146–458IU/l) and AST levels (700–1150IU/l). The exact peak AST and ALT values were not provided by Kucuk et al. Majority of the cases were below the age of 18 (88%, n = 15) and almost two-third (64.7%, n = 11) had supportive treatment with intravenous fluid administration. In cases reporting sex, there were more females than males (62.5%, 5/8 vs. 37.5%, 3/8). The timing between norovirus infection, as shown by the presence of symptoms, and the detection of elevated LFTs was not specified by Kucuk et al., however amongst the 4 patients in the study from Tsuge et al., on average, this was 6 days. For the 3 remaining studies, norovirus infection and detection of transaminitis was on the same day. The duration from onset of illness to recovery of symptoms was on average 10 days, but this value excludes the patients from Kucuk et al. [10] and Khayat et al. [13] because Kucuk et al. did not provide this information and Khayat et al. had a patient requiring 5 months to recover. This patient reported by Khayat et al., however, had an underlying disease. The duration from onset to recovery of the elevated LFT was available for 6 cases (excluding children with underlying conditions) at 22.5 days.

All cases recovered fully with no reported chronic liver disease or ongoing morbidity. There was no fatality reported.

Of all the cases with transaminitis, 17.6% (n = 3) had underlying medical conditions (comorbidities); one case previously had cholelithiasis and had undergone cholecystectomy, however the duration for recovery from norovirus induced acute hepatitis was not much longer than other cases. Two other cases previously had liver transplants; therefore, they were immunocompromised. Their duration for recovery of symptoms (14 days and 150 days respectively) and LFT results were significantly longer than other cases with an average of 375 days (Table 2).

**Table 1.** Description of study designs and reported norovirus induced transaminitis.

Study reference	Year of Publication	Country	Study design	Age group studied (years)	Data collection methods	Diagnostic method for norovirus	Timing between norovirus infection and hepatitis/transaminitis	Definition of hepatitis	Excluded other hepatotrophic causes (Y/N)
Nakajima H et al. [6]	2012	Japan (Tokyo)	Case Report	48	Retrospective routine laboratory investigations.	Detected by viral antigen check and confirmed by reverse transcription PCR.	Same day	Elevated LFTs	Y
Zenda T et al. [11]	2011	Japan (Ishikawa)	Case Report	56	Retrospective routine laboratory investigations.	Positive test for norovirus antigens in stool using immunochromatographic assay kits.	Same day	Elevated LFTs	Y
Kucuk O et al. [10]	2016	Turkey (Istanbul)	Retrospective hospital-based	0 to 17	Retrospective study from 2010 and 2013 of acute viral gastroenteritis.	Rapid antigen tests and PCR.	N/A	Elevated LFTs	Y
Tsuge et al. [12]	2010	Japan (Okayama)	Case series	1 to 7	Prospective study measuring LFTs and bloods in children presenting with gastroenteritis.	RT-PCR assay	Case 1: 5 days Case 2: 1 day Case 3: 6 days Case 4: 11 days	Elevated LFTs	Y
Khayat AA et al. [13]	2019	USA (Wisconsin)	Case series	3 and 8	Retrospective routine laboratory investigations.	Stool specimens for norovirus antigens.	Case 1 and 2: Same day	Elevated LFTs	Y

Notes: Abbreviations: FBC, full blood count; LFT, liver function test; USA, United States of America; PCR, polymerase chain reaction; RT-PCR, reverse transcription-polymerase chain reaction.

**Table 2.** Characteristics of studies included in the review.

Characteristics	Studies				
Study reference	Nakajima H et al. [6]	Zenda T et al. [11]	Kucuk O et al. [10]	Tsuge et al. [12]	Khayat AA et al. [13]
No. of patients	1	1	74	4	2
No. of cases	1	1	9	4	2
Sex (M/F)	F	F	N/A	Case 1: F; Case 2: F Case 3: M; Case 4: M	Case 1: F Case 2: M
Patient Age (yrs)	48	56	0–17	1–7	Case 1: 3 Case 2: 8
Symptoms	Abdominal pain and diarrhoea. Tenderness in epigastrium and right hypochondrium.	Abdominal cramps, vomiting, diarrhoea. Tenderness in left lower abdomen.	Vomiting, diarrhoea, and dehydration.	Vomiting, diarrhoea, dehydration, and fever with reduced bowel sounds. Case 2 had convulsions.	Anaemia, haematemesis, diarrhoea.
Peak AST (IU/l)	892	1150	45.6	794	700
Peak ALT (IU/l)	146	458	31.7	423	400
Peak GGT (IU/l)	N/A	73	N/A	N/A	N/A
Peak ALP (IU/l)	No rise	318	N/A	N/A	N/A
Other abnormal results	None	None	Hct 36.9, WCC 13510, CRP 9.7	Case 2: leucocytosis (WBC 13670IU/l) Case 3: hyponatraemia	Case 1 had normal liver biopsy.
Onset to recovery of symptoms (days)	7	4	N/A	Day 8, day 13, day 8 and day 18 in cases 1, 2, 3 and 4 respectively.	Case 1: 150; Case 2: 14.
Onset of illness to LFT recovery (days)	14	14	N/A	Case 1: 26; Case 2: 26;Case 3: 27; Case4: 28 .	Case 1: 480; Case 2: 270
Requiring hospitalisation/IV support	Hospitalisation	Hospitalisation	40 (54%) were hospitalized and 34 (46%) had an observed follow-up.	Cases 1–3 were hospitalized.	Hospitalised
Management	Supportive therapy	Bowel rest and intravenous rehydration.	IV rehydration	Intravenous fluids for cases 1-3 and oral/IV glycyrrhizin for all.	Case 1 had IV IVIG during their recovery from norovirus infection.
PMH	Cholelithiasis and cholecystectomy 4 years ago. Drinks alcohol.	Surgery for appendicitis, myoma uteri, gallstones (cholecystectomy)	N/A	N/A	Case 1 had a liver transplant due to hepatoblastoma. Case 2 had liver transplant due to congenital hepatic fibrosis and Caroli's disease.
Outcome of cases	Alive without chronic liver disease.	Alive without chronic liver disease.	N/A	Alive without chronic liver disease.	Alive without chronic liver disease.

Notes: Abbreviations: PMH, past medical history; IV, intravenous; Hct, haematocrit; WCC, white cell count; CRP, c-reactive protein; IVIG, immunoglobulins; M, male; F, female; N/A, not available.

## 4. Discussion

Acute hepatitis due to non-hepatotropic viruses such as EBV and HSV have been well described in the literature, but extra intestinal presentations of norovirus, such as disseminated coagulopathy and febrile convulsions, have been considered until recently, a rare phenomenon [7],[8]. A thorough review of the literature identified a very low reported rate of norovirus induced hepatitis, most likely because norovirus gastroenteritis is usually self-limiting and there has been no indication for invasive investigations, such as LFTs, in patients presenting with norovirus. There was a good clinical history of transaminitis, and acutely elevated liver enzymes as described by Zenda T and colleagues [11].

Interestingly, all published articles of norovirus induced acute hepatitis are less than a decade, which also support our findings. This review, in addition to other previously reported cases, would suggest that norovirus induced transaminitis is more common than previously reported in the literature. However, the mechanism of liver injury caused by norovirus remains unclear. One of the factors postulated is the release of pro-inflammatory cytokines induced by the viruses, which affects the inherent hepatic function, while other authors propose a host immune response with activated cytotoxic T cells affecting the hepatocytes [14],[15]. These presumptions are yet to be established by further studies.

Clinical presentations were similar in all cases, mainly vomiting and diarrhoea except one case that was complicated with convulsions [12], and another case with anaemia and haematemesis [13]. However, there seems to be an increase in abdominal tenderness in those with elevated transaminitis. Majority of the cases reported occurred in children and over two-thirds were treated in hospital with intravenous fluid administration. This was not surprising as children are likely to deteriorate faster with norovirus gastroenteritis compared to adults, but whether the liver of a child is more prone to extra intestinal manifestations of norovirus with acute hepatitis will require further investigations. Similar to the management of non-hepatotropic viruses, the treatment of acute hepatitis following norovirus gastroenteritis is mainly supportive, involving primarily rehydration and correction of electrolyte abnormalities. These patients are expected to recover, on average, between 1 to 3 days. However, for some individuals, particularly children, the elderly and individuals with underlying diseases, these symptoms can be severe and lead to dehydration, and in very rare cases even cause death [3],[4]. It is also important to note that many of these patients would have severe dehydration from vomiting and diarrhoea, which are both common symptoms of norovirus infection. As a result, elevated LFTs may result, however, this would not explain the lymphocytic infiltration seen using pathological investigations in the study from Khayat et al.

A longer recovery period in cases with norovirus induced hepatitis was observed. In another retrospective study in Japan, including 71 children with norovirus acute gastroenteritis, the mean duration of illness was twice as long in infants and these infants have a greater severity of illness [16]. However, concomitant extra intestinal manifestations were not explored, possibly because this is such a rare event and there was no robust evidence base to investigate children with suspected norovirus induced acute hepatitis, it is therefore prudent that children with protracted course of illness following norovirus gastroenteritis should be evaluated for possible extra intestinal manifestation, including acute hepatitis.

This study is not without limitations. Most of the included studies were either case reports or case series, which are generally of low evidence. In addition, 17.6% cases had an underlying liver disease, which will predispose them to liver diseases, and these were adjusted for in this review. In particular, Khayat et al. [13] describes two patients with liver transplants and who experienced norovirus induced hepatitis. Nevertheless, all cases were thoroughly investigated for other possible causes of liver dysfunction, and one of the articles completely excluded patients who were positive for other viruses [10]. All papers defined hepatitis as having elevated LFT results. Reassuringly, the elevated LFTs settled within three weeks and all cases recovered with no evidence of chronicity including fatality, but we observed a much longer recovery period in cases with underlying medical conditions.

## 5. Conclusion

Norovirus gastroenteritis is usually a self-limiting illness, requiring mainly supportive therapy. This review therefore highlights the need for clinicians to consider norovirus induced acute hepatitis, especially in children with protracted illness, and as a potential cause of elevated transaminases.
